# Multivariate classification of pain-evoked brain activity in temporomandibular disorder

**DOI:** 10.1097/PR9.0000000000000572

**Published:** 2016-09-30

**Authors:** Daniel E. Harper, Yash Shah, Eric Ichesco, Geoffrey E. Gerstner, Scott J. Peltier

**Affiliations:** a Chronic Pain and Fatigue Research Center, Department of Anesthesiology, School of Medicine, University of Michigan, Ann Arbor, MI, USA; b Functional MRI Laboratory, University of Michigan, Ann Arbor, MI, USA; c Department of Biologic and Materials Sciences, School of Dentistry, University of Michigan, Ann Arbor, MI, USA

**Keywords:** Neuroscience/neurobiology, Orofacial pain/TMD, Artificial intelligence, Brain function, Magnetic resonance imaging, Support vector machines

## Abstract

**Introduction:** Central nervous system factors are now understood to be important in the etiology of temporomandibular disorders (TMD), but knowledge concerning objective markers of central pathophysiology in TMD is lacking. Multivariate analysis techniques like support vector machines (SVMs) could generate important discoveries regarding the expression of pain centralization in TMD. Support vector machines can recognize patterns in “training” data and subsequently classify or predict new “test” data.

**Objectives**: We set out to detect the presence and location of experimental pressure pain and determine clinical status by applying SVMs to pain-evoked brain activity.

**Methods:** Functional magnetic resonance imaging was used to record brain activity evoked by subjectively equated noxious temporalis pressures in patients with TMD and controls. First, we trained an SVM to recognize when the evoked pain stimulus was on or off based on each individual's pain-evoked blood–oxygen–level–dependent (BOLD) signals. Next, an SVM was trained to distinguish between the BOLD response to temporalis-evoked pain vs thumb-evoked pain. Finally, an SVM attempted to determine clinical status based on temporalis-evoked BOLD.

**Results:** The on-versus-off accuracy in controls and patients was 83.3% and 85.1%, respectively, both significantly better than chance (ie, 50%). Accurate determination of experimental pain location was possible in patients with TMD (75%), but not in healthy subjects (55%). The determination of clinical status with temporalis-evoked BOLD (60%) failed to reach statistical significance.

**Conclusion:** The SVM accurately detected the presence of noxious temporalis pressure in patients with TMD despite the stimulus being colocalized with their ongoing clinical pain. The SVM's ability to determine the location of noxious pressure only in patients with TMD reveals somatotopic-dependent differences in central pain processing that could reflect regional variations in pain valuation.

## Abstract 2

Machine learning algorithms used evoked brain responses to detect noxious temporalis pressure in patients, and differentiated their brain responses depending on location of experimental pain.

## 1. Introduction

Temporomandibular disorders (TMD), which involve persistent pain in the cheek and jaw area of the face, have an estimated prevalence of approximately 11% in community samples.^34^ Temporomandibular disorder pain is a considerable impediment to quality of life as nearly 80% of TMD sufferers report regular discomfort eating and more than 40% report difficulty performing their jobs.^3^ The importance of central nervous system factors in TMD etiology has been well established. Neuroimaging has revealed structural,^13,37,38,44,56^ functional,^27,39,53,56^ and neurochemical^14^ aberrations in TMD. Multivariate analysis techniques, including support vector machines (SVMs), could help uncover the central mechanisms underlying altered pain processing in TMD, and determine whether these changes are related to ongoing clinical pain.

Support vector machines are machine learning algorithms that can first be trained to recognize patterns in data and can then be used to classify a separate set of data. These multivariate techniques have recently begun to be implemented to study pain processing in healthy individuals.^4,5,7,25,35,42,47,52^ For example, Wager et al.^52^ were able to determine when healthy subjects were experiencing noxious heat vs innocuous warmth based on each subject's pain-evoked brain activity, with 93% accuracy. They found that the brain regions driving this classification were some of those that are more generally known to be important for central pain processing, including the insula and anterior cingulate cortex (ACC). To extend this type of finding into the clinical realm, we first asked whether an SVM can accurately detect the presence of a noxious stimulus, based on its evoked brain response, when that stimulus is applied to a region where a person is experiencing ongoing clinical pain. In addition, we questioned whether an SVM can distinguish the location of noxious pressure based on evoked brain activity, which could give insight into location-specific somatotopic changes in pain processing because of regionally defined clinical pain. Multivariate analyses have also been used to determine clinical status in research settings. Three studies have used structural neuroimaging data (eg, gray matter volume) to classify pain patients vs controls, in irritable bowel syndrome (accuracy = 70%), chronic pelvic pain (73%), and chronic low back pain (76%),^1,30,51^ and there is already some evidence that stimulus-evoked brain responses can also be used to reliably determine clinical status in patients with chronic back pain (CBP) and fibromyalgia.^6,22^

In this study, we first used an SVM to identify when a noxious pressure was being applied to the temporalis muscle vs when it was not based on each individual's pain-evoked brain response. We hypothesized that it might be more difficult to detect the presence of experimentally evoked temporalis pain in patients with TMD compared with controls because of the patients' ongoing clinical pain. Next, we attempted to differentiate between the temporalis-evoked stimulation and noxious stimulation to a remote, asymptomatic area (ie, the thumb), using brain activity evoked by the 2 stimuli. We hypothesized that this would be possible in patients with TMD, but not in controls, because in the latter group neither area was clinically painful. Finally, we attempted to determine clinical status (ie, TMD vs control) based on temporalis-evoked brain activity.

## 2. Methods

### 2.1. Subjects

Ten patients with myofascial-type TMD (9 female) and 10 age-, sex-, and ethnicity-matched healthy control (HC) subjects were enrolled in the study. Results from other neuroimaging modality analyses have been reported elsewhere.^13,14,27^

All subjects with TMD were carefully examined by a dentist (GEG) with orofacial pain experience applying the research diagnostic criteria for the diagnosis of myofascial-type TMD (group 1a, 1b),^11^ and by an MD for medical history evaluation. Subjects fulfilling only the Group I myofascial pain criteria were eligible. Inclusion and exclusion criteria consisted of (1) presence of pain in the face, jaws, or temples greater than 1× per week; (2) presence of pain symptoms for greater than 3 months; (3) meeting the research diagnostic criteria criteria for myofascial pain group 1a, 1b; (4) no comorbidities of other chronic pain disorders (eg, fibromyalgia or irritable bowel syndrome). For HC subjects, the primary inclusion criterion was the absence of TMD pain, or facial pain less than 1× per week. Exclusion criteria for all subjects included physical impairment (eg, complete blindness, deafness, or paraplegia), or coexisting physical injury, any outstanding history of systemic or medical conditions, psychiatric illnesses, substance abuse within 2 years, and presence of head or neck pain other than masticatory myalgia. Nonsteroidal antiinflammatory drugs and other over-the-counter analgesics were allowed until 3 days before the pain and scanning visits. Medication overuse had been ruled out in all patients. All subjects were right handed. Because menstrual cycle phase can be coupled with pain symptoms,^31^ all female subjects participated in pain and imaging visits within 3 days of menstrual onset. All study participants gave written informed consent. The study protocol and informed consent documents were approved by the University of Michigan Institutional Review Board.

### 2.2. Clinical pain and behavioral data

Clinical pain was assessed using the Short-Form McGill Pain Questionnaire (SF-MPQ),^36^ which consists of a visual analog scale anchored on the left with “No Pain” and on the right with “Worst Possible Pain.” A second component of the SF-MPQ consisted of 11 sensory and 4 affective descriptors that are rated as either “none,” “mild,” “moderate,” or “severe,” by subjects. Jaw function status was measured using the Jaw Functional Limitation Scale,^40^ which asks subjects about their limitations during the past month. Mood was evaluated using the State-Trait Personality Inventory.^48^ The State-Trait Personality Inventory is a self-report tool that measures anxiety and depression separately on a 4-point intensity scale.

Categorical scores that occurred in some of the above instruments were converted to numerical scores. For all instruments, numerical scores of individual items or sums across items were calculated and used in analyses.

### 2.3. Experimental pain data

Experimental pressure pain data were collected for all subjects. Pressure pain testing was conducted on the left anterior temporalis and the left thumbnail (as a control area with no clinical pain in either group) using the multiple random staircase method (MRS) as previously described.^16,19,23,27^ Pain ratings were recorded using a 21-box numerical descriptor scale,^41^ which was constructed from previously determined verbal descriptors.^15,17,18^ Pressure pain testing resulted in 2 MRS levels each for the thumbnail and anterior temporalis: medium pain (pressures that elicited ratings of 7–8 on the descriptor box scale), and high pain (pressures that elicited ratings of 13–14 on the descriptor box scale).

Clinical pain, experimental pain, and demographic data were analyzed for significant differences between groups in SPSS, version 21, using the Mann–Whitney *U* test because of the relatively small sample size. Differences were deemed significant at *P* < 0.01 after a Bonferroni correction for multiple comparisons.

### 2.4. Neuroimaging data acquisition, preprocessing, and analysis

Magnetic resonance imaging was performed on a 3.0 Tesla GE Signa scanner (LX [VH3] release, Neuro-optimized gradients). Evoked pressure–pain data were acquired using a T2*-weighted spiral sequence (repetition time = 2.5 seconds, echo time = 30 milliseconds, flip angle = 90°, matrix size 64 × 64 mm with 48 slices, field of view = 22 cm, and 3.44 × 3.44 × 3 mm voxels), using a birdcage transmit-receive radio frequency coil. A high resolution structural image (repetition time = 1400 milliseconds, echo time = 1.8 milliseconds, flip angle = 15°, field of view = 256 × 256, yielding 124 sagittal slices with a defined voxel size of 1 × 1 × 1.2 mm) was acquired using T1-weighted spoiled gradient echo inversion recovery sequence for each subject. Inspection of individual T1 images revealed no gross morphologic abnormalities for any subject. Each subject underwent 2 functional magnetic resonance imaging–evoked pressure pain scans: during the first, pressure was applied to the left thumbnail and in the second it was applied to the left anterior temporalis, as previously described.^19^ We chose to conduct the thumb run first to minimize any carryover effects that might have occurred after stimulation of a clinically painful area in patients with TMD. Pressures eliciting high and medium pain, previously determined in the behavioral session of the MRS testing, were used to evoke painful responses during scans for the thumbnail and anterior temporalis, respectively, so as to measure Blood–oxygen–level–dependent (BOLD) activations. Each scan lasted 10 minutes. Pressures were applied in a pseudo-random fashion and were interleaved with an “off” condition where no pressure was applied. Both the thumbnail and face runs each contained a total of 12 pain blocks (6 medium, 6 high; each block 25 seconds in duration) and 12 off blocks (each block 25 seconds in duration).^21,27^

Functional magnetic resonance imaging data for the thumbnail and face evoked scans were preprocessed and analyzed using FSL (www.fmrib.ox.ac.us/fsl) and SPM (http://www.fil.ion.ucl.ac.uk/spm/) software packages. SPM version 8 was run in MATLAB 7.5b (Mathworks, Sherborn, MA). Preprocessing steps included slice-timing correction, motion correction, normalization to Montreal Neurological Institute (MNI) space, and spatial smoothing (8 mm FWHM Gaussian kernel). Subject head motion was assessed by evaluating 3 translations and 3 rotations for each scan. Translational thresholds were set to ±2 mm, and rotation thresholds were set to ±1°. A subject was to be excluded from the analysis if head motion exceeded either of the thresholds in 1 of the 6 dimensions, though none of the participants exceeded them.

To rule out the potential that the SVM was detecting differences in head motion between groups and/or conditions, we assessed average stimulation-induced head movement for the thumb and temporalis pain. Mean head motion (6 parameters total) was calculated for each participant. Separate analysis of variances were conducted for translational and rotational movement parameters.

### 2.5. Support vector machine analysis

An SVM analysis was performed using the LIBSVM toolbox, within MATLAB, version 3.18.^8^ A linear kernel with parameter C = 1 was implemented (no improvement was found doing a C parameter line search).

For the first analysis, examining prediction of pain vs rest in the functional runs, the input data were the BOLD images for the pain runs, with each volume labeled as pain or rest (medium and high pain were both labeled as pain). To minimize signal decay effects from prolonged pain stimuli,^26^ the data from the first half of each block (pain or rest) was used. Training was then performed on the first half of the run, with testing performed on the second half. Prediction accuracy was defined as the number of test volumes correctly predicted.

For the second analysis looking at classification of thumb vs face pain, the input data were the general linear model (GLM) pain vs rest contrast maps for each run, with each map labeled as thumb or face. Leave-one-run-out cross-validation was used to calculate classification accuracies and predicted values. The 2 groups were analyzed separately.

For the classification of subjects with TMD vs controls, the input data were the GLM pain vs rest contrast maps for each subject from the temporalis run, with each map labeled as TMD or control. Leave-one-subject-out cross-validation was used to calculate classification accuracies and predicted values.

For all analyses, SVM model weights were averaged across all instances to investigate spatial distribution of the significant weights driving the models. Permutation testing was performed to generate significance levels for the model weights, by permuting the treatment labels 100 times for each leave-one-out instance, resulting in 2000 model weight instances for each voxel location, allowing significance to be calculated by the number of times a model weight occurred in the histogram. Significant values were overlaid on reference anatomy, and the contrast values of the most significant areas were plotted to examine their relationship to the multivariate pattern.

## 3. Results

### 3.1. Demographics, clinical pain, and behavioral data

Complete behavioral results are presented in Table 1. Patients with temporomandibular disorder were found to have significantly more clinical pain than the HC group based on the SF-MPQ sensory component, the total score, and the VAS. The Jaw Functional Limitation Scale revealed significant functional limitations in the mastication and mobility, but not verb/emot, of patients with TMD. After Bonferroni correction, the 2 groups did not differ significantly with respect to mood on any of the measures tested. When comparing experimental pain between the 2 groups, patients with TMD did not differ significantly from controls in pressure pain sensitivity on the thumb or the face.

**Table 1:**
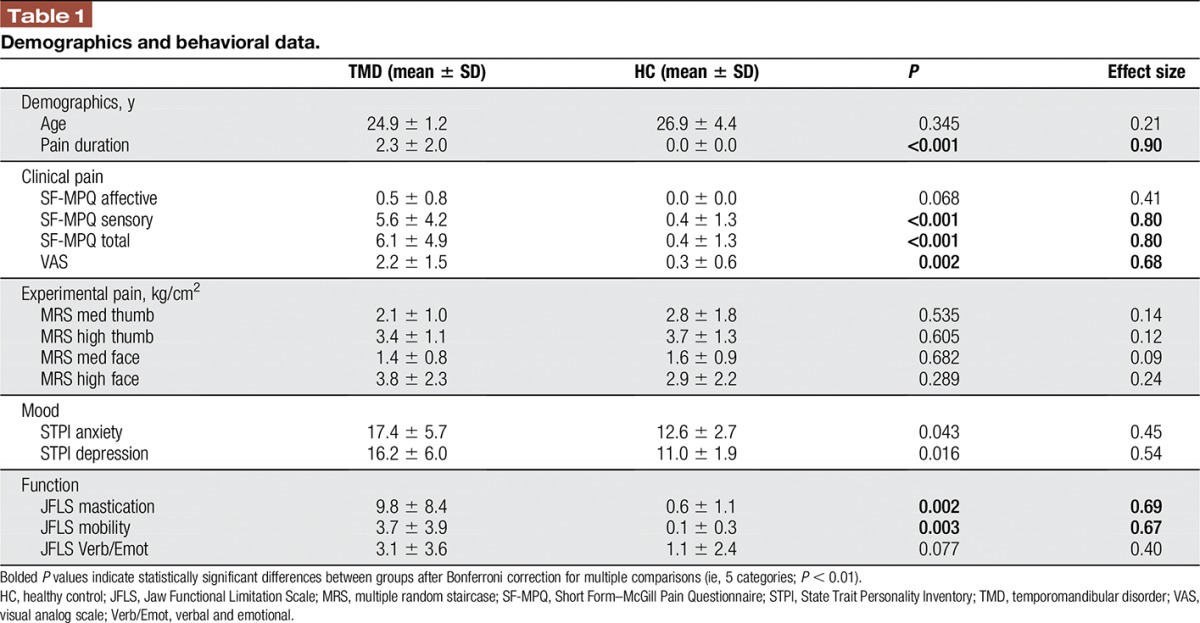
Demographics and behavioral data.

### 3.2. Head motion

Two 2 × 2 × 3 repeated-measures analysis of variances (site [thumb vs temporalis] × group [HC vs TMD] × dimension [x y z]) revealed no significant main effects of site (F_(1,18)_ = 0.43, *P* = 0.52 and F_(1,18)_ = 0.38, *P* = 0.55) or group (F_(1,18)_ = 0.66, *P* = 0.43 and F_(1,18)_ = 0.02, *P* = 0.89) for translational or rotational movement, respectively. Likewise, the interactions between group and stimulation site were not significant for translational (F_(1,18)_ = 1.16, *P* = 0.30) or rotational (F_(1,18)_ = 0.67, *P* = 0.42) movement. Thus, head motion did not differ between groups, between stimulation sites, or between sites differentially for the 2 groups.

### 3.3. Support vector machine prediction of evoked temporalis pain vs rest

**Figure 1.**
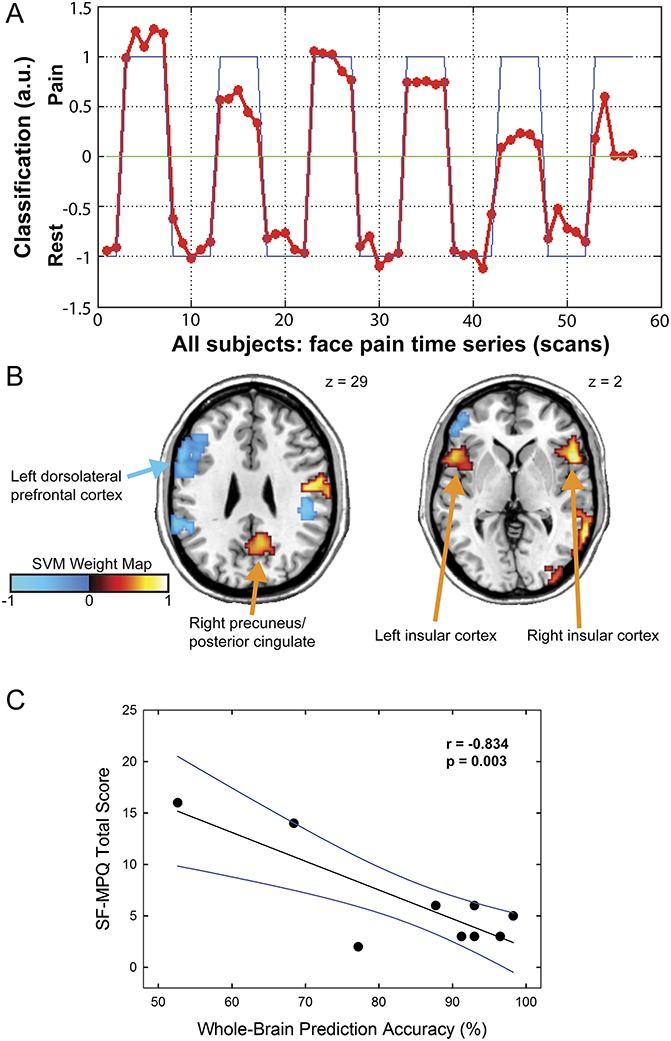
Classification of pain vs off. (A) The mean prediction plot for temporalis-evoked pain vs rest for all subjects. Ideal prediction in blue, actual prediction in red, and decision plane at 0 in green. (B) Mean weight vector maps for face pain vs rest in all subjects. Pain > rest in orange, rest > pain in blue. Brighter colors indicate higher predictive value. (C) Negative correlation in patients with TMD between classification accuracy and SF-MPQ Total Score. Confidence intervals of 95% are shown in blue. Two patients have identical coordinates (x = 92.98, y = 3). SF-MPQ, Short form McGill Pain Questionnaire; SVM, support vector machine; TMD, temporomandibular disorder.

By training on the first half and testing on the second half of each run, and vice versa; the average prediction accuracy was significantly better than chance in all cases (*P* < 0.0001), 84.2 (±14)% for the 2 groups combined, 85.1 (±14.6)% in subjects with TMD, and 83.3 (±15)% in controls (Table 2 for individual prediction accuracies). Figure 1A shows the average prediction results as a function of scan number.

**Table 2:**
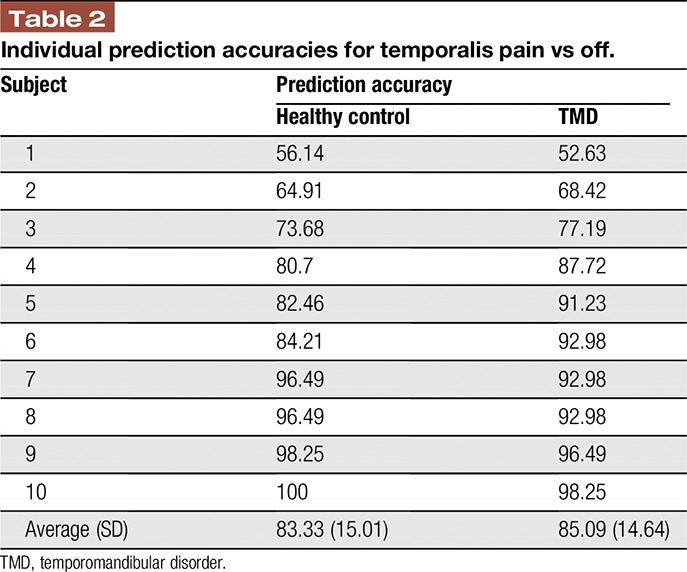
Individual prediction accuracies for temporalis pain vs off.

The average weight vector maps, depicting the regions of the brain that were most predictive in the SVM classifying pain vs off, for all subjects combined are shown in Figure 1B. Significant regions included some classical pain processing regions like bilateral insula, ACC, and precuneus. A complete list of significant clusters is provided in Table 3.

**Table 3:**
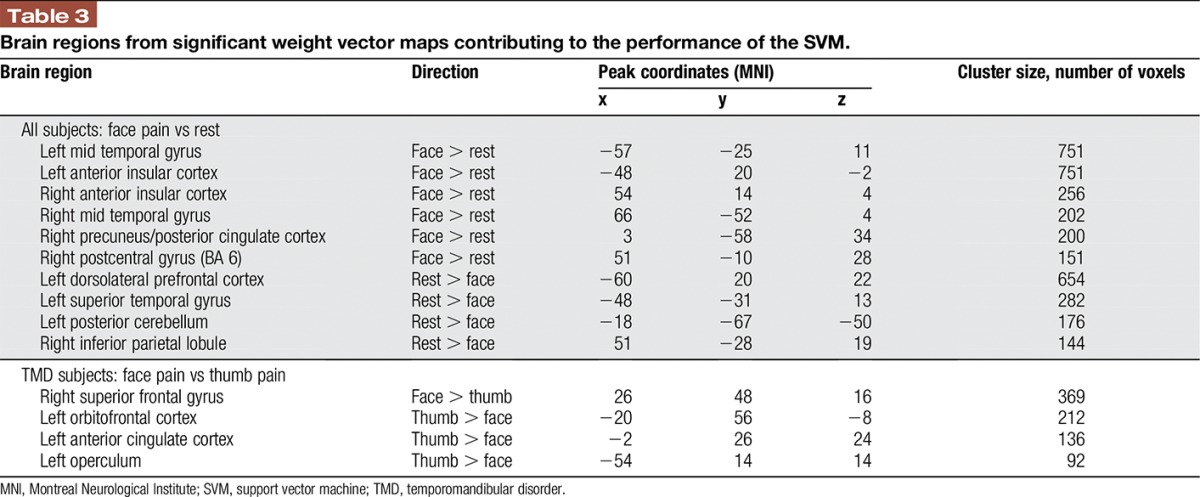
Brain regions from significant weight vector maps contributing to the performance of the SVM.

In the TMD group, there was a significant negative correlation between clinical pain level (MPQ total) and percent correct, meaning that the SVM was less effective in discriminating between temporalis-evoked pain and rest in individuals with higher ongoing clinical pain (Fig. 1C).

### 3.4. Support vector machine prediction of face pain vs thumb pain in patients with temporomandibular disorder and controls

**Figure 2.**
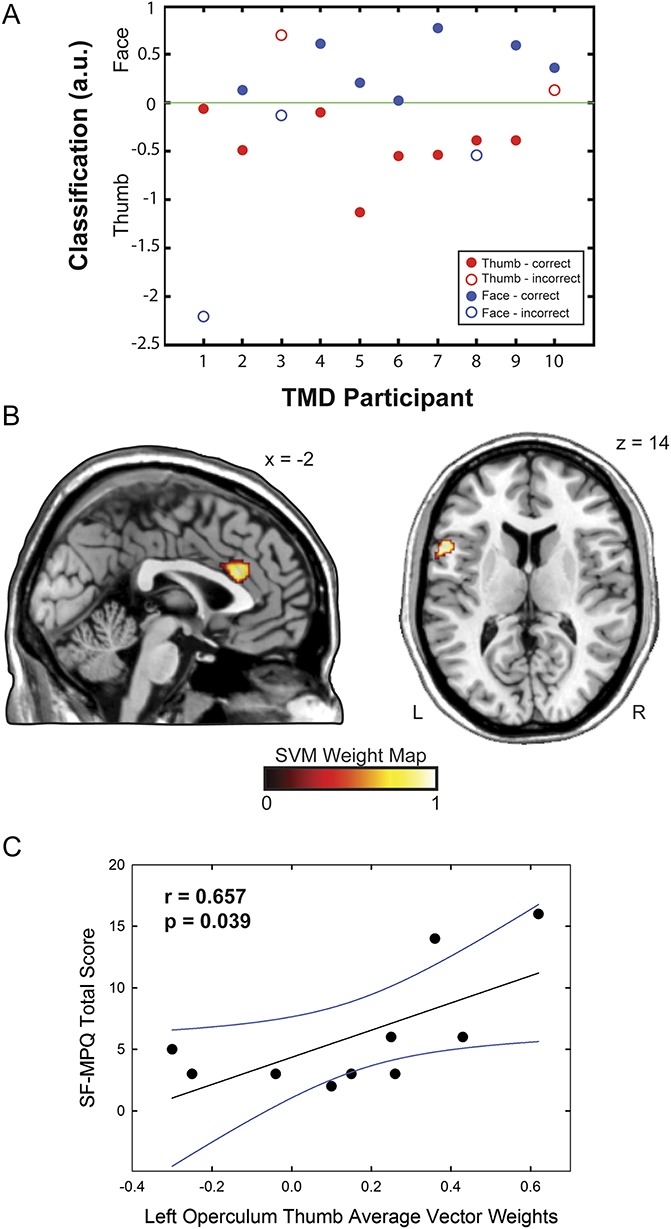
Classification of evoked pain location in patients with TMD. (A) Classification accuracy for temporalis vs thumb pain. Predictions for the thumb are in red, predictions for the face are in blue, and the decision plane at 0 is in green. (B) Mean weight vector maps for temporalis pain vs thumb pain. Regions within the anterior cingulate cortex and operculum depict thumb >face. Brighter colors indicate higher predictive value. (C) Correlation between mean vector weights and SF-MPQ total scores. Confidence intervals of 95% are shown in blue. L, left; R, right; SF-MPQ, Short Form McGill Pain Questionnaire; SVM, support vector machine; TMD, temporomandibular disorder.

Using a leave-one-out classification on the GLM contrasts for both the face pain run and thumb pain run in subjects with TMD, prediction accuracy was 75%, which was significantly better than chance (*P* = 0.02) (Fig. 2A). In contrast, the SVM was not able to differentiate the brain response to thumb pain vs temporalis pain in HC subjects. Accuracy in this case was 55%, no better than chance performance (*P* = 0.25). The average weight vector maps, depicting the most predictive regions, for patients with TMD are shown in Figure 2B. There was a significant positive correlation between operculum BOLD response to thumb pain and MPQ score. The more clinical pain a patient reported, the higher the opercular activity was in response to thumb pain (Fig. 2C).

In addition, there was a trend toward a significant relationship between TMD clinical pain level and the accuracy of differentiating experimental pain location. Patients were grouped based on whether the algorithm correctly identified both runs, which it did for 6 patients. Mean MPQ total score for them was 8.0 (±5.6), whereas it was 3.3 (±1.3) in the 4 patients for whom 0 or 1 location was correctly identified. A Mann–Whitney *U* test showed that the difference in clinical pain between these groups trended toward being statistically significant (z = 1.77; *P* = 0.077; effect size = 0.56), where higher clinical pain was associated with better prediction accuracy of pain location.

### 3.5. Support vector machine prediction of patients with temporomandibular disorder vs controls

Using a leave-one-out classification on the temporalis pain run GLM contrasts, the average prediction accuracy was 60%. This only approached statistical significance (*P* = 0.10).

## 4. Discussion

In this study, patients with TMD and HCs were subjected to noxious pressure on the temporalis muscle, an area affected with clinical pain in most patients with TMD, and on the thumb, an unaffected remote area that was used as a control. The BOLD response to noxious pressure applied to the temporalis was analyzed using an SVM to make several classifications, including (1) the presence (vs absence) of the noxious stimulus, (2) noxious pressure applied to the temporalis vs the thumb, and (3) clinical status as a patient or a control. The results showed an excellent ability to determine when noxious pressure was being applied to the temporalis, in both patients and controls. The ability to distinguish between temporalis- and thumb-evoked pain was significantly higher in patients with TMD; the SVM was unable to distinguish between the signals evoked from the 2 locations in control subjects. Finally, we were unable to predict clinical status using temporalis pain–evoked BOLD responses, though the accuracy approached significance.

### 4.1. Detection of pain and its location

Multivariate pattern analysis techniques have been used to study pain processing in healthy subjects, using both functional magnetic resonance imaging^4,5,7,35,42,52^ and electroencephalography.^25,47^ These studies have all used either thermal- or laser-evoked noxious stimulation, but their detection rates for those types of pain (ranging from 61% to 93%) were similar to our detection of pressure pain (84%). Previous multivariate studies also found similar brain areas that drove the classification of pain, such as the insula, somatosensory, and cingulate cortices.^4,5,35,52^ The fact that the SVM was able to detect a signal against the underlying backdrop of colocalized chronic pain in the patients with TMD, and using regions similar to those detected in healthy subjects, suggests that the induced neural signals were strong enough to still enable accurate classification. However, the correlation between clinical pain and SVM detection accuracy in patients suggests that the results might differ within TMD. The relative inability to detect experimental pain in those with high clinical pain would be expected if the experimental pain had an increased carry-over effect into the off blocks due to increased aftersensations or sensitization,^43^ which would have introduced additional noise to the BOLD contrast for evoked pain.

To our knowledge, this study is the first to attempt to determine the location of a noxious stimulus using SVM, by inclusion of both a temporalis- and a thumb-evoked pain. Physical stimulus intensities were tailored to accommodate differences in experimental pain sensitivity both within- (ie, location) and between-individuals, which should have normalized the magnitude of evoked cortical activity.^10^ The SVM was able to accurately detect the difference between evoked thumb and face pain in patients with TMD, but not in controls, indicating differences in the way experimental pain is processed when applied to a symptomatic region of the body. These differences included decreased responses to temporalis-evoked pain in the left orbitofrontal cortex, ACC, and operculum. These regions have been shown to be involved in the cognitive valuation of pain,^29,55^ which might be expected to differ depending on whether experimental pain is applied to a clinically painful region of the body. This result indicates that the location of a stimulus can be assessed using machine learning by its cognitive valuation, even when the mode of stimulation (eg, noxious pressure) and its perceived intensity are held constant.

Whereas higher clinical pain made detection of temporalis-evoked pain less accurate in the TMD group, it trended toward making differentiation of pain location more accurate. Although speculative, it may be that clinical pain's regional effect on the valuation of experimental pain is proportional to its intensity. Larger samples should permit a more rigorous analysis of how SVMs can be used to identify differences in central pain processing within groups of patients with TMD.

### 4.2. Detection of clinical status

There has also been some success of classifying patients with chronic pain vs control subjects using structural and functional MRI. Differences in regional gray matter volume have been used to distinguish cohorts of chronic back and pelvic pain and irritable bowel syndrome from controls at accuracies ranging from 70% to 76%.^1,30,51^ Perhaps most similar to this study's attempt to classify clinically using functional data, Callan et al.^6^ were able to differentiate patients with CBP from controls with 92% accuracy, using a sparse logistic regression and data based on BOLD responses to noxious electrical stimulation applied to the back. It is unclear whether their superior classification accuracy was due to the differences in methods (eg, electrical vs pressure stimulation, sparse logistic regression vs SVM, high vs low sample size, etc) or differences between TMD and CBP. More research will be needed in this regard.

Finally, there is also a recent study demonstrating that patient classification can also be achieved using nonnoxious stimulus-evoked brain responses. Here, BOLD responses to a flashing checkerboard (perceived to be unpleasant by many) were able to differentiate patients from controls with 82% accuracy using SVM.^22^ Furthermore, in a smaller subset of patients who underwent a crossover pregabalin/placebo treatment, degree of right insula activation by the visual stimulus was positively correlated with responsiveness to pregabalin, and classified drug vs placebo with 82% accuracy. Presumably, in centralized pain like FM, generalized hypervigilance and hypersensitivity across sensory modalities is reflected in functional brain differences that reliably distinguish patients from controls. It remains to be seen whether these findings are replicated in other conditions with centralized pain.

Temporomandibular disorder is a highly heterogeneous group of disorders involving the TMJ and surrounding structures, and there is ample evidence that many patients have centralized changes in pain processing,^9,14,24,28,32,33,45^ often marked by widespread hyperalgesia. In this study, there were no significant differences between the groups in pressure pain sensitivity or pain-evoked BOLD response for a location remote from the TMJ (the thumb), suggesting that pain centralization was not causing widespread hyperalgesia in this sample of patients. This is likely due to our exclusion of patients with TMD whose pain was not well localized to the TMJ and surrounding muscles and individuals with a variety of comorbid conditions, who would be expected to have more pronounced pain centralization.^2^

### 4.3. Limitations

This study's small sample size increases the possibility of model overfit, poor generalizability, and type I and II errors, so the results must be interpreted with caution. Only 2 of our patients with TMD had high levels of clinical pain, and the reported relationships (eg, Figs. 1C, 2C) within TMD are not significant with those subjects removed. These relationships will need to be examined again in larger samples, including patients with TMD who have higher levels of clinical pain and more evidence of pain centralization.^20^

### 4.4. Future directions

However, despite its small sample size and a TMD cohort that possessed relatively low levels of clinical pain, this study revealed patient-specific differences in the brain response to noxious temporalis pressure. The fact that SVMs were able to detect differences between patients with HCs and TMD using noxious pressure stimuli that were tailored to produce equal perceived pain intensity across the 2 groups and locations shows the sensitivity of using SVM to assess and categorize pain-evoked brain activity.

Because TMD serves as a label for where the pain is perceived more than an explanation of its etiology, it is important to understand interpatient variance in degree of pain centralization, but this is rarely considered when choosing treatment course for TMD. Many providers perform a peripherally focused treatment such as an occlusal splint or physiotherapeutic techniques,^54^ whereas the central changes that are often apparent in TMD and other chronic pain conditions are left untreated. To its credit, however, the TMD Diagnostic Criteria^46^ do provide an axis on which biopsychosocial variables can be assessed, and some studies show improvement in prognosis for those who score high on this axis when a centrally acting treatment (eg, cognitive behavioral therapy) is implemented along with usual standard of care.^12,49,50^ Further research is needed to determine whether SVMs can help identify subtypes of patients who have been diagnosed with TMD and whose pain might have a more peripheral vs a central etiology, which could be clinically useful; however, the gold standard of TMD diagnosis will continue to be self-reported pain. Pain biomarkers, where found, should never be used to replace or undermine the experience of the patient. Nevertheless, even in the immediate future, many patients will feel that their symptoms are vindicated by the presence of objective central findings as reported in studies such as this one.

### 4.5. Conclusions

This study provides a first step toward applying machine learning algorithms to TMD and shows that pain may be processed differently, despite controlling for its intensity, depending on whether it is applied to a clinically painful area.

## Conflict of interest statement

The authors have no conflicts of interest to declare.

This study was supported by NIH Grant DE018528 to G. E. Gerstner. D. E. Harper is supported by NIH grant K12-DE023574.

D. E. Harper and Y. Shah contributed equally to this article. G. E. Gerstner and S. J. Peltier contributed equally to this article.
